# 806 Autologous Skin Cell Suspension Demonstrates Successful Outcomes in the Treatment of Burn and Wound Sites

**DOI:** 10.1093/jbcr/irae036.346

**Published:** 2024-04-17

**Authors:** Brian Draper, Cindy Austin, Oluwafolaranmi Sodade

**Affiliations:** Mercy Hospital - Springfield, Springfield, MO; Mercy Hospital, Nixa, MO; Mercy Hospital, Springfield, MO; Mercy Hospital - Springfield, Springfield, MO; Mercy Hospital, Nixa, MO; Mercy Hospital, Springfield, MO; Mercy Hospital - Springfield, Springfield, MO; Mercy Hospital, Nixa, MO; Mercy Hospital, Springfield, MO

## Abstract

**Introduction:**

A new treatment paradigm increasing among burn and wound providers is the application of Autologous Skin Cell Suspension (ASCS). This system is a point-of-care therapy spraying on the patient's own cells promoting epidermal regeneration while reducing the amount of skin harvested for donor sites. Literature reports on the use of ASCS but there is a paucity of data describing the application of ASCS with temporal dermal substitutes and donor sites. The purpose of this study is to evaluate complex wound sites due to burns and other etiologies treated with ASCS solely or in combination with wound care applications. Also, a review of ASCS applied over donor sites is reported. We hypothesize the application of ASCS on complex wounds, burns and off-label usage on donor sites to demonstrate acceptable outcomes.

**Methods:**

A retrospective chart review of burn and wound patients (N=53) treated with ASCS between 2019-2022 at a tertiary care center. Data was extracted from the electronic medical record. Treatments evaluated included ASCS only, ASCS with or without autologous split-thickness skin graft (STSG), and polyurethane bioabsorbable matrix (PBM) placement prior to ASCS. Wound healing results were collected on the off-label use of ASCS applied to donor sites. Each individual wound site was measured (cm2) and failure rate was calculated based on size of dermal substrate or graft loss. Statistical analysis software was used to summarize data which included descriptive demographics, wound type/size, graft fail size, percent total burn, adjunct procedures, comorbidities, systemic/ local wound infection rate, complications, length of stay, and overall patient outcome summary.

**Results:**

A total of 53 burn and wound patients were included, 46(87%) and 7(13%), respectively. The mean age was 44.3 (SD 20.6) years with 35(66%) patients being male. The mean total burn surface area was 30% (SD 23.8%). The most common comorbidities were hypertension, coronary artery disease and kidney disease. Highest complication was infection n=26 (49%), 11 local, 7 systemic and 8 both. A total of 577 injury sites were evaluated. The fail rate for PBM+STSG+ASCS, STSG+ASCS, ASCS+ non-donor site, and ASCS on donor site was (2.9%, 0.4%, 0.0%, 0.5%), respectively. No complications were reported with ASCS on donor sites. The percent fail of STSG’s without ASCS was 6.6% and reduced to 0.4% when used in combination with STSG+ASCS.

**Conclusions:**

The findings of this study data demonstrate the successful application of ASCS even in the presence of infection on burn and complex wounds used alone or in conjunction with PBM. The results support the off-label use of ASCS on donor sites.

**Applicability of Research to Practice:**

This study demonstrates the effectiveness of ASCS on wounds as well as the use on donor sites. This study adds to the growing literature evaluating the use of ASCS on burn wounds.

